# Insulin Resistance and Bone Mineral Density: A Comprehensive Examination Using UK Biobank Data

**DOI:** 10.3390/healthcare12242502

**Published:** 2024-12-11

**Authors:** Yu-Tun Hung, Tsong-Han Yu, Javad Alizargar

**Affiliations:** 1 Department of Orthopedics Surgery, Department of Medicine, Hualien Armed Forces General Hospital, Hualien 971, Taiwan; doc20834@mail.ndmctsgh.edu.tw; 2 Department of Orthopedics, Tri-Service General Hospital, National Defense Medical Center, Taipei City 114, Taiwan; 3 School of Nursing, National Taipei University of Nursing and Health Sciences, Taipei City 112, Taiwan

**Keywords:** insulin resistance, bone mineral density, TyG index, osteoporosis, propensity score matching, UK Biobank

## Abstract

**Purpose:** The association between insulin resistance (IR) and bone mineral density (BMD) remains contentious. The aim of this study is to assess the predictive capability of the Triglyceride and Glucose (TyG) index concerning changes in bone mineral density, encompassing both deterioration and improvement. **Methods:** This study analyzed data from the UK Biobank, encompassing 2527 participants after exclusions. Logistic models and ANOVA were employed, with propensity score matching addressing the effects of age, BMI, and sex. The TyG index was calculated using this formula: Ln (triglyceride [mg/dL] × glucose [mg/dL]/2). **Results:** Initially, a positive correlation was observed between the TyG index and BMD measures. However, upon adjustment for age, sex, and BMI, this association lost significance. Propensity score matching further indicated no inverse relationship between the TyG index and osteoporosis development. **Conclusions:** Although the TyG index demonstrated a positive correlation with BMD, caution is warranted due to potential confounding by age, sex, and BMI. Notably, the TyG index alone did not predict changes in T-score or osteoporosis status.

## 1. Introduction

Bone mineral density (BMD) denotes the amount of minerals present within bone tissue and serves as a quantifiable gauge of bone mass and resilience. A decline in BMD beyond a specific level can precipitate osteoporosis and elevate the likelihood of fractures [1].

It is projected that the prevalence of osteoporosis among various demographic groups differs based on measurements at lumbar, femoral neck, and total hip sites. For instance, among US Caucasian women, the estimated prevalence rates stand at 15.8%, 20.4%, and 15.2%, respectively. Conversely, for US black women, the prevalence rates are lower, at 6.7%, 7.8%, and 7.9%. Similarly, among Chinese women, the estimated rates are 7.5%, 7.5%, and 6.7%. Among US black men, the rates are 1.8%, 5.7%, and 3.3%, and among Chinese men, they are 2.0%, 3.8%, and 3.4% [2].

Osteoporosis is a chronic metabolic bone disease marked by a reduced bone mass and microarchitectural degradation, leading to a decreased BMD and increased fracture risk. It results from an imbalance in osteoclast and osteoblast activity, causing accelerated bone resorption or delayed bone formation. BMD, assessed through DXA, is a key clinical indicator for diagnosing osteoporosis, which continues to rise in prevalence due to increasing life expectancies. Metabolic factors like glucose and lipid metabolism have gained attention for their role in bone health. Abnormalities in glucolipid metabolism, such as elevated triglycerides and total cholesterol, have been linked to increased osteoporosis risk, though findings on HDL-C, LDL-C, and BMD remain inconsistent. Bone loss advances silently and gradually, often without noticeable symptoms until a serious fracture occurs. Hence, comprehending the factors impacting bone mineral density (BMD) holds paramount importance. Various elements of daily life can influence BMD, including dietary habits such as the consumption of fatty foods and engagement in physical activity. Research suggests that elevated levels of high-density lipoprotein cholesterol (HDL-C) might affect osteoclast activation or function through the stimulation of inflammatory responses. Notably, exercise plays a crucial role in improving bone tissue by imposing mechanical stress, which stimulates cellular processes involving osteoblasts, osteoclasts, and osteocytes. This activity promotes bone remodeling and generally leads to positive changes in bone mineral density (BMD), strengthening bone structure and reducing the risk of fractures [3].

Metabolic Syndrome (MetS) is a cluster of cardiometabolic risk factors, including central obesity, insulin resistance, hypertension, elevated triglycerides, high fasting glucose levels, and reduced HDL-C levels [4]. Studies conducted on non-diabetic adults in the United States revealed a direct relationship between the Metabolic Syndrome Insulin Resistance (METS-IR) score and bone mineral density (BMD) levels. Specifically, an increment of one unit in METS-IR significantly elevates total femoral and spinal BMD [5].

The relationship between insulin resistance (IR) and bone mineral density (BMD) is contentious. Some studies have linked a lower BMD with IR [6], while some other investigations contradicted this by suggesting that lifelong IR might actually elevate BMD. Moreover, the impact of IR on bone mass appears to vary across genders and bone locations in non-diabetic adults. These inconsistencies likely stem from multifaceted factors, with variations in study populations emerging as a particularly noteworthy consideration [7].

Recent research often uses the TyG index to assess insulin resistance, with findings indicating a connection between elevated TyG index and increased risks of adverse cardiac and cerebrovascular events in STEMI (ST-Elevation Myocardial Infarction) patients undergoing PCI (Percutaneous Coronary Intervention). Moreover, there is a proportional rise in the risk of ischemic stroke with increasing TyG index levels. Utilizing this index in large-scale databases could be cost-effective and feasible, given that many databases contain data on plasma glucose and triglyceride levels, allowing for the exploration of various relationships based on this index [8]. No study has explored whether the computed insulin resistance index (TyG index) can predict an individual’s osteoporosis status or be linked to declines in bone mineral density (BMD).

The aim of this study is to assess the predictive capability of the TyG index concerning changes in bone mineral density, encompassing both deterioration and improvement.

## 2. Methods

**Figure 1:**
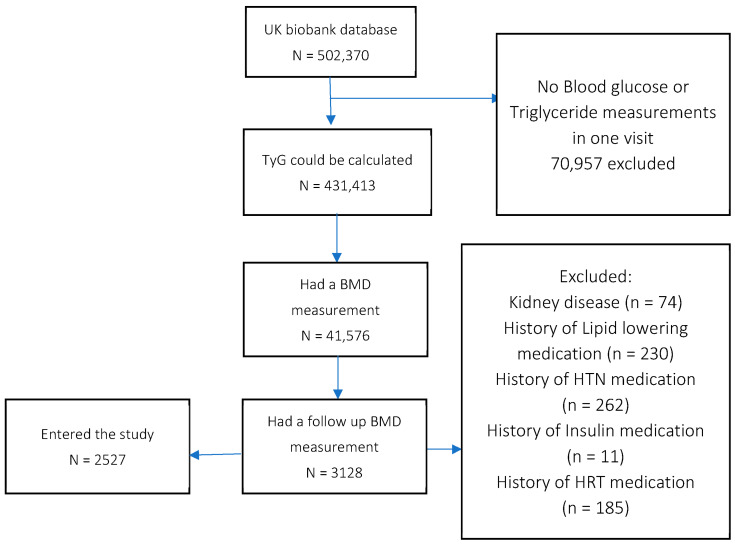
Flowchart of the study.

Using data extracted from the UK Biobank, we evaluated information from 502,370 individuals based on inclusion and exclusion criteria. Among these, 431,413 individuals had the necessary data for calculating their TyG index from their blood glucose and triglyceride levels. Additionally, 41,567 participants had data on bone mineral density (BMD) measurements, with 3128 returning for follow-up BMD assessments between 2014 and 2019. After excluding individuals with kidney disease (ICD-10 codes N17-N19) and those using medications for lipidemia, hypertension (HTN), diabetes mellitus (DM), or hormone replacement therapy (HRT), data from 2527 individuals were included in the study. A flowchart of the study is outlined below (Figure 1).

The study utilized data on bone mineral density (BMD) in the ribs and pelvis (gr/cm^2^), as well as the total T-scores of the femur and L1–L4 spine, alongside the individual’s overall T-score, to compute the change in BMD between the baseline and revisit periods. The TyG index was calculated as the natural logarithm (Ln) of the product of plasma glucose level and triglyceride using this formula: Ln (triglyceride [mg/dL] × glucose [mg/dL]/2).

Descriptive statistics, along with univariate and multivariate logistic models employing ANOVA tests, were utilized for data analysis. SAS 9.4 was the software used for data analysis.

Osteoporosis is defined as a T-score below −2.5 standard deviations (SDs). A T-score between −1 and −2.5 is considered indicative of a low bone mineral density (BMD), while a T-score above −1 is classified as a normal BMD [9]. The worsening of osteoporosis status was defined as either the development of osteoporosis (total T-score less than −1 for individuals without osteoporosis initially) or a decrease in T-score to less than −2.5 for individuals with an initial T-score between −1 and −2.5.

To investigate whether the discrepancy in TyG levels between individuals experiencing a worsening of osteoporotic status and those maintaining the same status arises from differences in age, sex, and BMI, a propensity score matching approach was adopted. This involved matching individuals on a one-to-one basis based on age, BMI, and sex. Subsequently, logistic regression was conducted to assess the impact of these factors on TyG levels among individuals with the same sex.

## 3. Results

### 3.1. Descriptive Statistics

Table 1 displays the descriptive statistics of the individual data included in our study.

### 3.2. Correlation Analysis Between TyG and Osteoporosis

In Table 2, TyG is divided into four quartiles, and each quartile was tested against various measures of osteoporosis to examine their correlation. The Spearman’s rank correlation coefficient, along with the corresponding *p*-values and the number of observations, is presented.

### 3.3. Analysis of TyG Levels in Different Groups of Participants

#### 3.3.1. Comparison of TyG Levels Between Individuals with Decreased BMD and Those Without Decreased BMD

Individuals with a respective BMD or T-score lower than the baseline were categorized as having a “Decreased BMD”. This binary variable was employed in an ANOVA test to examine differences in TyG levels between individuals with and without a decrease in BMD (Table 3).

#### 3.3.2. Analysis of TyG Discrepancy Between Individuals with Worsening Osteoporosis Status and Those Without, Using GLM Model

The differences in tyg levels between individuals with worsening osteoporosis status and those without can be found in Table 4.

### 3.4. Association Between TyG and BMD

To investigate the association between TyG and BMD, both univariate and multivariate analyses were conducted on the study participants. Logistic regression was employed for the dependent variable due to its non-normal distribution. Two comprehensive sets of regression analyses were carried out to determine if the TyG levels in an individual predict decreases in T-score and the worsening or emergence of osteoporosis. All models controlled for age, sex, and BMI in the multivariate analysis.

#### 3.4.1. Predictive Capability of TyG for Decreasing T-Score

Individuals with a T-score lower than the baseline were categorized as having a “Decreased T-Score”, and this binary variable was utilized as the dependent variable in logistic regression analysis. The results of these sets of analyses are presented in Table 5.

#### 3.4.2. Propensity Score-Matched Logistic Regression Analysis

The results from the univariate logistic regression predicting worsening osteoporotic status using T-scores from the three regions can be seen in Table 6.

**Figure 2:**
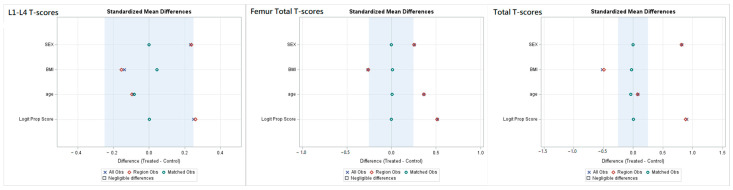
Standardized mean differences in T-scores from three regions after propensity score matching; **left**: L1–L4 T scores; **middle**: femur total T score; **right**: total T score.

The standardized mean differences following propensity score matching in T-scores from three regions are illustrated in Figure 2.

**Figure 3:**
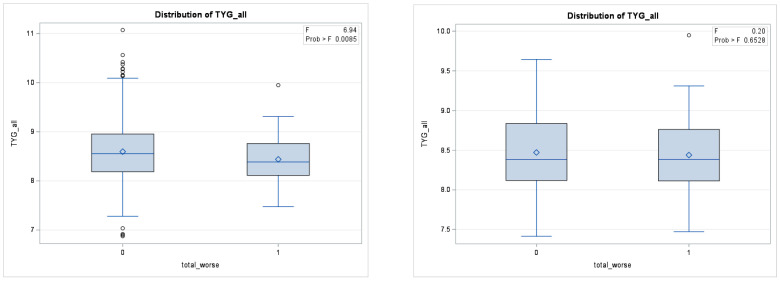
Difference in TyG Levels between groups with worsening T-scores before matching (**Left**) and after matching (**Right**).

**Figure 4:**
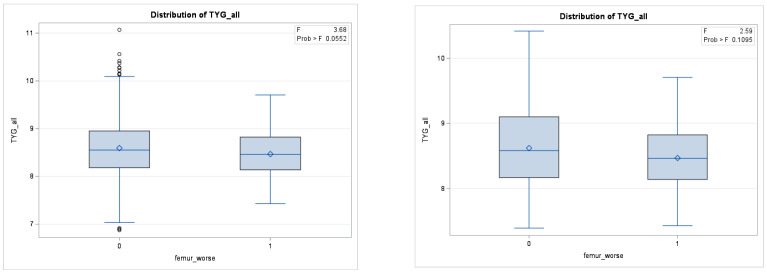
Difference in TyG Levels between groups with worsening total T-score in the femur before matching (**left**) and after matching (**right**).

**Figure 5:**
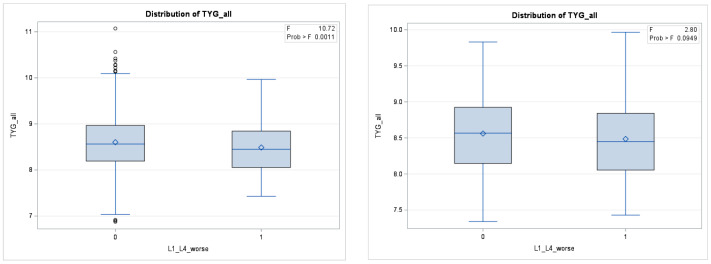
Difference in TyG Levels between groups with worsening L1-L4 T-scores before matching (**left**) and after matching (**right**).

A comparison of the TyG index before and after propensity score matching in the three regions can be found in Figure 3, Figure 4 and Figure 5.

## 4. Discussion

The TyG index demonstrated a notable positive correlation with BMD measures, and individuals with higher TyG levels tended to have stable BMD. However, after adjusting for age, sex, and BMI, these findings lost significance. Notably, our study is the first to utilize propensity score-matched controls to mitigate the influence of age, BMI, and sex on individuals’ osteoporosis status during follow-up. This matching approach revealed that the TyG index is not inversely associated with the development of osteoporosis.

Some studies have indicated both negative and positive associations between insulin resistance indices and osteoporosis. For instance, Zhuo et al. (2023) conducted an extensive investigation into the relationship between insulin resistance (IR), as assessed by the TyG index, and bone health. Their study encompassed both a cross-sectional analysis involving 788,247 participants and a longitudinal study with 8770 participants. They observed a robust positive correlation between the TyG index and low bone mass, osteoporosis, or both, in both study designs, even after adjusting for confounding variables (all *p* < 0.001). Furthermore, baseline TyG index levels significantly predicted the subsequent development of low bone mass, osteoporosis, or both during the follow-up period. Adjusting for confounders, hazard ratios (HRs) were 1.56 (95% CI: 1.25, 1.93, *p* < 0.05), 1.66 (95% CI: 1.06, 2.59, *p* < 0.05), and 1.55 (95% CI: 1.27, 1.88, *p* < 0.05) for a low bone mass, osteoporosis, or both, respectively. These findings underscore the significant association between IR, as measured by the TyG index, and an increased risk of low bone mass and osteoporosis. However, the generalizability of this study may be constrained due to the high prevalence of a low bone mass or osteoporosis (45.64%) and obesity and metabolic syndrome (46.81% and 35.08%, respectively) among their participants. Moreover, the study did not exclude individuals using insulin or lipid-lowering medications, a factor of significance given the substantial number of diabetic and metabolic syndrome patients. Given that the TyG index relies on plasma glucose and triglyceride levels for calculation, these aspects may raise questions about the study’s conclusions [10].

Zhan et al. (2023) used NHANES data to study the TyG index’s relationship with bone mineral density (BMD). They divided subjects into four TyG index groups and applied linear regression, constructing four multivariable models. They used restricted cubic spline and piecewise linear regression for analysis and conducted subgroup assessments. Their findings showed significant variations across US adults in their TyG index quartiles. A negative correlation between the TyG index and lumbar spine BMD was noted. Comparing the lowest (Q1) to the highest (Q4) TyG index quartile, lumbar spine BMD’s β value was −0.008 (95% CI: −0.017, 0). A nonlinear association between the TyG index and lumbar spine BMD was identified, with a threshold was determined through spline analyses. Above this threshold, a significant negative association was observed (β = −0.042, 95% CI: −0.059, −0.024). No significant interactions were found in subgroup analyses based on age, gender, diabetes, BMI, or medication usage. Similar patterns were observed for total and subtotal bone density. Although this study also shows some associations, they did not exclude the patients that using insulin, lipid-lowering, or hormonal treatment medications. As these drugs can easily change the calculations of TyG, the final interpretation can be spurious [11].

Wen et al. (2022) examined 832 non-diabetic individuals, finding significant associations between TyG-BMI, bone mineral density (BMD), and fracture risk. The TyG-BMI combines the Triglyceride–Glucose (TyG) index, a marker of insulin resistance derived from fasting triglycerides and glucose, with Body Mass Index (BMI), a measure of adiposity. This composite index reflects the interplay between metabolic dysfunction and obesity, offering a practical tool for assessing metabolic health. It is increasingly used in research to explore associations with conditions like cardiovascular disease, diabetes, and cancer, providing a non-invasive alternative to direct measures of insulin resistance. The formula for calculating the TyG-BMI is typically TyG-BMI = TyG index × BMI. TyG-BMI correlated positively with BMD and negatively with fracture risk in men and, after adjustments, in women. The results of this association could be because of BMI only. Further studies on the fracture risk are necessary to find out the association between the TyG index and fracture risk [12].

Tian et al. (2024) analyzed NHANES data (2011–2018) with 5456 participants, finding a positive correlation between TyG and BMD, adjusting for covariates. This is explainable as insulin resistance (IR) stimulates insulin secretion, leading to an increased bone mineral density (BMD) by promoting osteoblast proliferation and inhibiting osteoclast activity. Additionally, IR affects bone metabolism through inflammatory responses and estrogen levels. Inflammatory mediators and estrogen influence osteoclast and osteoblast activity, affecting bone remodeling. This reciprocal relationship implies a cycle wherein IR affects bone metabolism, and bone health in turn affects glucose metabolism. However, other factors, particularly obesity, also play a significant role in these metabolic interactions [13].

Xuan (2024) examined 1182 non-diabetic men aged 50 years or older using NHANES data. They employed a two-piecewise linear regression model to analyze TyG-BMI’s relationship with femoral neck (FN) bone mineral density (BMD), exploring threshold effects. The results revealed a positive association between TyG-BMI and FN BMD, with a nonlinear pattern across TyG-BMI tertiles. Subgroup analyses confirmed this positive association across different groups. This association may not be applicable to our study since incorporating BMI into the TyG index may not accurately predict TyG index outcomes. Additionally, it is essential to separately investigate the relationship between the TyG index and BMD to ensure a comprehensive analysis [14].

TyG index is an indirect proxy measure of insulin resistance. To measure the insulin resistance for large scale datasets and as a screening test, a proxy measure can be used. The calculation of insulin resistance using the insulin level of the individuals yields an index for insulin resistance named Homa-ir. There are some reports in the literature that also investigated insulin resistance with BMD using homa-ir. Fu et al. (2022) [15] explored the connections between insulin resistance, β-cell secretion, bone mineral density (BMD), and osteoporosis using National Health and Nutrition Examination Survey data. They analyzed BMD data from 5292 participants assessed via dual-energy x-ray absorptiometry. Their findings indicated that the relationship between HOMA-β and BMD/osteoporosis varied with increasing HOMA-IR levels. Specifically, HOMA-β showed a negative association with osteoporosis when HOMA-IR was less than 2, but this association was not significant when HOMA-IR was 2 or higher. Upon employing a regression model that accounted for age, sex, and BMI, the correlation between HOMA-IR and BMD vanished. These results align with our own investigation.

The period known as the menopause transition, or perimenopause, is linked with a faster decline in bone mineral density (BMD), heightening the likelihood of osteoporosis and fractures. Shieh et al. [16] investigated how insulin resistance (measured by HOMA-IR) relates to changes in lumbar spine (LS) and femoral neck (FN) bone mineral density (BMD) across various menopausal stages. They discovered a two-phase connection: lower HOMA-IR levels corresponded to a slower BMD decline, while higher levels showed no significant link. Moreover, alterations in HOMA-IR mirrored changes in BMD, suggesting that reducing insulin resistance may protect BMD, while increased resistance may lead to BMD loss. These patterns were consistent in postmenopausal women but less pronounced during the menopause transition. In their study, the significant findings exhibited notably low regression coefficients following adjustments for age and body composition measures, with marginal *p*-values. These associations could potentially diminish if the participants were matched.

Some studies have suggested beneficial effects of insulin resistance on bone mineral density (BMD). For instance, Ye et al. (2023) [17] examined 437 nondiabetic postmenopausal women, investigating bone density alongside insulin resistance markers. They observed a higher insulin resistance among women with a normal bone density compared to those with osteoporosis or osteopenia. Moreover, a higher insulin resistance correlated with increased bone density, even after adjusting for BMI. Furthermore, follicle-stimulating hormone (FSH) showed a negative association with bone density, mediated by insulin resistance markers. This implies that insulin resistance may play a role in mediating the FSH-induced suppression of bone density, independent of BMI. While their findings are intriguing and scientifically sound, the study lacked individual follow-up, and its results represent a cross-sectional analysis, thus limiting their ability to establish temporality.

Wang et al. (2021) [18] analyzed lumbar bone mineral density (BMD) and insulin resistance, measured using HOMA-IR (CP), in 234 patients with Type 2 Diabetes Mellitus (T2DM). Their findings revealed a significant, nonlinear association between HOMA-IR (CP) and osteoporosis in females after adjusting for covariates, with a HOMA-IR (CP) > 4.00 markedly increasing osteoporosis risk. Notably, gender played a critical role in the relationship between insulin resistance and osteoporosis risk in T2DM patients. Their study also highlighted that the association between insulin resistance and BMD diminishes after adjusting for age and BMI in multivariate models, aligning with our findings. While HOMA-IR is a more comprehensive index for assessing insulin resistance, its reliance on insulin serum levels—tests not routinely conducted in clinical practice—limits its use as a screening tool.

Shin et al. (2014) [6] studied 3113 Korean men, measuring their bone mineral density (BMD) and evaluating insulin resistance using HOMA-IR. They found that higher HOMA-IR and fasting insulin levels were associated with a lower BMD. Interestingly, the relationship between fasting insulin levels and BMD varied based on the insulin resistance level, while a low insulin resistance showed a positive association, higher insulin resistance correlated with reduced BMD, with significance increasing with insulin resistance severity. While some aspects of their conclusion align with our findings, it is essential to note that their study exclusively focuses on men and employs a cross-sectional design.

The observational nature of our study and its reliance on retrospective data limit the ability to draw definitive causal conclusions regarding the associations between lipid profiles, the TyG index, and melanoma incidence. These limitations are inherent in non-experimental designs and must be considered when interpreting our findings. However, we mitigated potential biases through rigorous statistical adjustments for key confounding factors such as age, sex, and BMI, which strengthens the reliability of our results. While our findings challenge conclusions drawn from some previous studies, our aim is not to refute their validity but rather to contribute new evidence that highlights the complex interplay of metabolic and lipid-related factors in melanoma. Future prospective studies or randomized controlled trials would be valuable to confirm and expand upon these associations.

We excluded individuals with a history of lipid-lowering, antihypertensive, insulin, or hormone replacement therapy (HRT) medications, as well as those with kidney disease, to ensure the accuracy of our analysis. These factors can significantly influence blood glucose and triglyceride levels, directly affecting the TyG index and potentially introducing confounding bias. By excluding these groups, we aimed to minimize external influences on TyG levels, ensuring that the observed associations with osteoporotic status were primarily attributable to age, sex, and BMI, as per the objectives of our study.

The TyG index, while practical and widely used as a surrogate for insulin resistance, has inherent limitations. It relies solely on fasting glucose and triglyceride levels, which may introduce variability due to dietary or metabolic factors. Although less comprehensive than HOMA-IR, which includes insulin levels, the TyG index remains advantageous for large-scale studies due to its reliance on routine clinical tests. Despite adjusting for key confounders like age, sex, and BMI, residual confounding or measurement bias cannot be entirely excluded, highlighting the need for future studies incorporating direct insulin resistance measures.

Our research exclusively utilizes data from participants with available follow-up imaging, with TyG calculations conducted before their initial imaging visit, implying a temporal relationship. However, the retrospective nature of the data collection presents limitations. Changes in patients’ conditions between visits, such as the initiation of insulin or lipid-lowering medications or the development of kidney disease, were not accounted for and could introduce bias. Prospective national cohorts and longitudinal studies could offer valuable insights into TyG’s role in BMD. Though costlier and more challenging, utilizing methods like HOMA-IR and insulin clamp for insulin resistance measurement could enhance study accuracy. While propensity score matching aids in making patient groups comparable, it cannot replace the need for randomized controlled trials (RCTs). RCTs are essential to validate our findings.

## 5. Conclusions

While the TyG index shows a notable positive correlation with BMD, it is essential to consider potential confounding factors such as age, sex, and BMI. TyG does not independently predict changes in T-score or an individuals’ osteoporosis status.

## Figures and Tables

**Table 1:** Descriptive statistics of study participants’ data.

Variable	Total N	Means	SD	N	%
Sex (Female)	-	-	-	1040	41.16
Age	2527	52.39	7.43	-	-
BMI	2527	26.13	3.87	-	-
TyG	2527	8.58	0.54	-	-
Baseline Ribs BMD	2527	0.86	0.13	-	-
Revisit Ribs BMD	2527	0.84	0.14	-	-
Baseline Pelvis BMD	2527	1.02	0.15	-	-
Revisit Pelvis BMD	2527	1.00	0.15	-	-
Baseline L1-L4 T score	2454	−0.06	1.53	-	-
Revisit L1-L4 T score	2426	−0.18	1.57	-	-
Baseline Femur Total T score	2475	0.36	0.62	-	-
Revisit Femur Total T score	2475	0.27	0.62	-	-
Baseline Total T score	2527	0.74	1.18	-	-
Revisit Total T score	2527	0.55	1.19	-	-

**Table 2:** Correlation between TyG quartiles and osteoporosis measures using spearman’s rank correlation coefficient.

Variables		Revisit Pelvis BMD	Baseline Pelvis BMD	Revisit Ribs BMD	Baseline Ribs BMD	Revisit L1–L4 T Score	Baseline L1–L4 T Score	Revisit Total T Score	Baseline Total T Score	Baseline Femur Total T Score	Revisit Femur Total T Score
TyG (Q1)	r	0.04	0.03	0.09	0.06	0.10	0.06	0.04	0.02	0.04	0.07
	*p*	0.35	0.48	0.03	0.13	0.01	0.12	0.27	0.58	0.33	0.10
	N	631.00	631.00	631.00	631.00	610.00	615.00	631.00	631.00	617.00	620.00
TyG (Q2)	r	0.18	0.17	0.26	0.23	0.17	0.15	0.16	0.15	0.14	0.15
	*p*	<0.01	<0.01	<0.01	<0.01	<0.01	<0.01	<0.01	<0.01	<0.01	<0.01
	N	632.00	632.00	632.00	632.00	602.00	611.00	632.00	632.00	617.00	617.00
TyG (Q3)	r	0.05	0.05	0.10	0.12	0.06	0.09	0.05	0.05	0.07	0.05
	*p*	0.17	0.17	0.01	0.00	0.17	0.02	0.21	0.17	0.09	0.19
	N	633.00	633.00	633.00	633.00	602.00	618.00	633.00	633.00	624.00	616.00
TyG (Q4)	r	0.06	0.04	0.11	0.12	0.00	0.00	0.02	−0.01	0.05	0.04
	*p*	0.14	0.28	0.01	0.00	1.00	0.95	0.68	0.75	0.25	0.26
	N	631.00	631.00	631.00	631.00	612.00	610.00	631.00	631.00	617.00	622.00
**TyG**	r	0.22	0.20	0.39	0.37	0.20	0.16	0.15	0.11	0.16	0.19
	*p*	<0.0001	<0.0001	<0.0001	<0.0001	<0.0001	<0.0001	<0.0001	<0.0001	<0.0001	<0.0001
	N	2527.00	2527.00	2527.00	2527.00	2426.00	2454.00	2527.00	2527.00	2475.00	2475.00

**Table 3:** Comparison of TyG Levels between individuals with and without a decreased bone mineral density (BMD).

Measure of BMD		N	Means	SD	F Value	*p* Value
Ribs BMD	Decreased BMD	1795	8.56	0.54	13.51	<0.01
	No Decreased BMD	732	8.65	0.54		
Pelvis BMD	Decreased BMD	1936	8.56	0.54	14.98	<0.01
	No Decreased BMD	591	8.66	0.56		
L1–L4 T score	Decreased BMD	1610	8.53	0.53	50.27	<0.01
	No Decreased BMD	917	8.69	0.56		
Femur Total T score	Decreased BMD	1932	8.57	0.54	10.70	<0.01
	No Decreased BMD	595	8.65	0.56		
Total T score	Decreased BMD	1938	8.56	0.54	20.91	<0.01
	No Decreased BMD	589	8.67	0.55		

BMD = bone mineral density.

**Table 4:** Descriptive differences in TyG Levels between individuals with worsening osteoporosis status and those without.

Measure of BMD		N	Means	SD	F Value	*p* Value
L1-L4 T score	Worse OP status	274	8.48	0.53	10.72	<0.01
	same OP status	2253	8.60	0.54		
Femur Total T score	Worse OP status	73	8.46	0.50	3.68	0.05
	same OP status	2454	8.59	0.54		
Total T score	Worse OP status	88	8.43	0.45	6.94	<0.01
	same OP status	2439	8.59	0.55		

OP = osteoporosis.

**Table 5:** Logistic regression analysis of TyG’s predictive ability for decreasing t-score.

Dependent Variable		Beta Coefficient **	*p* Value	OR	CI	
					UL	LL
Decrease in Ribs BMD	univariate	−0.29	<0.01	0.74	0.63	0.87
	Multivariate *	−0.03	0.69	0.96	0.80	1.15
Decrease in Pelvis BMD	univariate	−0.32	<0.01	0.72	0.6	0.85
	Multivariate *	−0.12	0.2	0.88	0.72	1.06
Decrease in L1–L4 T score	univariate	−0.53	<0.01	0.58	0.5	0.68
	Multivariate *	−0.30	<0.01	0.73	0.61	0.87
Decrease in Femur total T score	univariate	−0.27	<0.01	0.75	0.64	0.89
	Multivariate *	−0.17	0.07	0.83	0.69	1.01
Decrease in the total T score	univariate	−0.38	<0.01	0.67	0.57	0.80
	Multivariate *	−0.10	0.29	0.9	0.74	1.09

* Controlled for age, sex, BMI; ** of TyG index; UL and LL: upper and lower limits.

**Table 6:** Univariate logistic regression for predicting worsening of osteoporotic status based on T-scores in three regions.

	Beta Coefficient **	*p* Value	OR	CI	
				UL	LL
L1–L4 T score	−0.27	0.09	0.76	0.55	1.04
Femur total T score	−0.47	0.11	0.62	0.34	1.11
total T score	−0.14	0.65	0.86	0.46	1.6

** of TyG index; UL and LL: upper and lower limits.

## Data Availability

The UK Biobank is an open access resource accessible to qualified researchers. Researchers with a legitimate interest can request access to the UK Biobank dataset by registering and submitting an application at http://www.ukbiobank.ac.uk/register-apply/ (accessed on 1 January 2023).
